# Supplementary material for: Effects of Different Processing Methods Based on Different Drying Conditions on the Active Ingredients of Salvia miltiorrhiza Bunge
Source: Molecules. 2022 Jul 29;27(15):4860. doi: 10.3390/molecules27154860 (PMC9369592; doi:10.3390/molecules27154860)
Supplement: Supplementary file 1 [file molecules-27-04860-s001.zip › molecules-1822341-supplementary.pdf]

Supplementary Materials

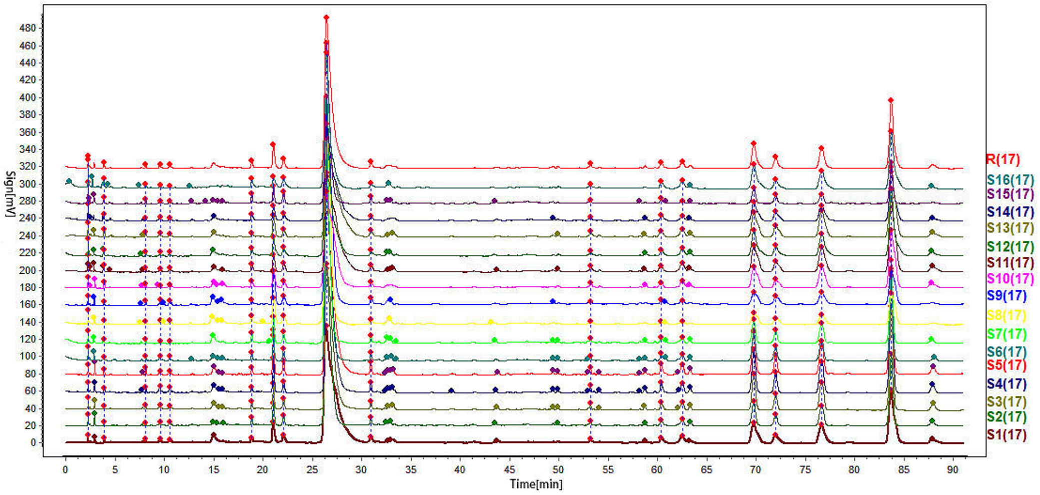

Figure S1. HPLC fingerprints of all active ingredients of 16 batches of different processing *S. miltiorrhiza.*

Table S1. The relative retention time of the common peaks of the 16 batches of *S. miltiorrhiza* with different processing.

No.	The Relative Retention Time																RSD (%)
	**S1**	**S2**	**S3**	**S4**	**S5**	**S6**	**S7**	**S8**	**S9**	**S10**	**S11**	**S12**	**S13**	**S14**	**S15**	**S16**	
1	0.026	0.026	0.026	0.026	0.026	0.027	0.026	0.027	0.027	0.026	0.026	0.027	0.027	0.027	0.027	0.026	1.640
2	0.045	0.047	0.046	0.045	0.046	0.047	0.047	0.046	0.046	0.046	0.046	0.046	0.046	0.047	0.047	0.046	1.422
3	0.095	0.097	0.096	0.096	0.096	0.098	0.098	0.096	0.097	0.097	0.096	0.098	0.096	0.096	0.098	0.100	1.315
4	0.112	0.114	0.113	0.113	0.112	0.115	0.115	0.116	0.113	0.115	0.112	0.116	0.112	0.113	0.115	0.117	1.473
5	0.125	0.126	0.125	0.125	0.124	0.126	0.127	0.129	0.127	0.126	0.125	0.128	0.125	0.125	0.126	0.128	1.106
6	0.226	0.226	0.226	0.226	0.227	0.225	0.225	0.224	0.225	0.226	0.226	0.225	0.227	0.226	0.225	0.224	0.416
7	0.251	0.253	0.252	0.251	0.252	0.252	0.252	0.252	0.252	0.253	0.252	0.252	0.252	0.252	0.252	0.252	0.144
8	0.265	0.265	0.266	0.265	0.266	0.264	0.263	0.261	0.263	0.265	0.265	0.262	0.265	0.265	0.263	0.261	0.605
9	0.321	0.318	0.322	0.319	0.322	0.315	0.312	0.304	0.309	0.320	0.323	0.310	0.323	0.320	0.314	0.306	1.991
10	0.371	0.371	0.371	0.371	0.372	0.369	0.369	0.367	0.369	0.371	0.371	0.369	0.371	0.370	0.369	0.367	0.393
11	0.636	0.636	0.635	0.635	0.635	0.635	0.635	0.636	0.636	0.637	0.636	0.636	0.636	0.636	0.636	0.636	0.079
12	0.721	0.722	0.721	0.720	0.721	0.720	0.721	0.721	0.721	0.722	0.723	0.721	0.723	0.721	0.721	0.721	0.092
13	0.747	0.748	0.747	0.746	0.746	0.747	0.747	0.747	0.748	0.747	0.748	0.747	0.747	0.747	0.747	0.747	0.061
14	0.834	0.832	0.834	0.834	0.834	0.834	0.834	0.833	0.833	0.832	0.834	0.833	0.834	0.834	0.834	0.834	0.069
15	0.860	0.859	0.860	0.860	0.860	0.860	0.860	0.860	0.860	0.859	0.860	0.860	0.860	0.860	0.860	0.860	0.037
16	0.916	0.915	0.916	0.916	0.916	0.916	0.916	0.916	0.917	0.916	0.916	0.916	0.916	0.916	0.916	0.916	0.036
17	1.000	1.000	1.000	1.000	1.000	1.000	1.000	1.000	1.000	1.000	1.000	1.000	1.000	1.000	1.000	1.000	0.000

Table S2. The relative peak area of the common peaks of the 16 batches of S. miltiorrhiza with different processing.

No.	The Relative Peak Area																RSD (%)
	S1	S2	S3	S4	S5	S6	S7	S8	S9	S10	S11	S12	S13	S14	S15	S16	
1	0.022	0.032	0.019	0.015	0.016	0.026	0.027	0.045	0.035	0.028	0.020	0.035	0.021	0.031	0.101	0.062	64.680
2	0.015	0.012	0.009	0.008	0.012	0.014	0.012	0.022	0.014	0.020	0.008	0.009	0.016	0.017	0.039	0.016	49.146
3	0.016	0.011	0.010	0.011	0.017	0.013	0.017	0.032	0.037	0.015	0.011	0.013	0.013	0.013	0.033	0.014	49.456
4	0.021	0.022	0.017	0.023	0.018	0.024	0.014	0.022	0.022	0.011	0.014	0.017	0.016	0.023	0.034	0.016	28.560
5	0.019	0.027	0.012	0.021	0.018	0.025	0.022	0.049	0.044	0.015	0.011	0.022	0.010	0.013	0.021	0.011	52.177
6	0.031	0.047	0.028	0.016	0.024	0.040	0.056	0.083	0.039	0.031	0.035	0.031	0.018	0.042	0.088	0.055	49.862
7	0.170	0.160	0.117	0.129	0.183	0.171	0.180	0.326	0.395	0.139	0.099	0.119	0.124	0.134	0.240	0.081	48.226
8	0.074	0.080	0.054	0.051	0.078	0.085	0.087	0.124	0.118	0.061	0.061	0.064	0.053	0.088	0.182	0.089	39.971
9	3.971	3.809	2.893	3.196	3.457	3.929	4.279	7.166	6.935	2.812	2.598	3.060	2.533	3.485	6.343	2.657	38.632
10	0.045	0.051	0.034	0.033	0.050	0.049	0.048	0.079	0.078	0.044	0.038	0.049	0.041	0.041	0.087	0.031	33.987
11	0.037	0.027	0.028	0.029	0.031	0.030	0.030	0.031	0.031	0.039	0.041	0.019	0.042	0.038	0.035	0.019	21.554
12	0.066	0.052	0.048	0.057	0.063	0.070	0.063	0.064	0.067	0.058	0.057	0.061	0.057	0.083	0.090	0.082	17.880
13	0.108	0.079	0.077	0.075	0.081	0.079	0.074	0.062	0.064	0.096	0.094	0.091	0.087	0.087	0.063	0.103	16.803
14	0.408	0.355	0.362	0.365	0.383	0.405	0.350	0.394	0.353	0.386	0.387	0.408	0.353	0.417	0.441	0.451	8.118
15	0.146	0.147	0.141	0.155	0.145	0.181	0.170	0.189	0.199	0.133	0.131	0.138	0.144	0.178	0.213	0.146	15.785
16	0.373	0.353	0.344	0.330	0.326	0.329	0.358	0.298	0.320	0.392	0.355	0.364	0.375	0.324	0.299	0.349	7.772
17	1.000	1.000	1.000	1.000	1.000	1.000	1.000	1.000	1.000	1.000	1.000	1.000	1.000	1.000	1.000	1.000	0.000

Table S3. The similarity results of the common peaks of the 16 batches of S. miltiorrhiza with different processing.

No.	S1	S2	S3	S4	S5	S6	S7	S8	S9	S10	S11	S12	S13	S14	S15	S16	Sr.
S1	1.000	1.000	0.995	0.998	0.999	1.000	1.000	0.992	0.992	0.994	0.991	0.997	0.990	0.999	0.994	0.991	1.000
S2	1.000	1.000	0.996	0.999	0.999	1.000	1.000	0.991	0.992	0.995	0.992	0.997	0.991	0.999	0.994	0.991	1.000
S3	0.995	0.996	1.000	0.999	0.998	0.995	0.992	0.975	0.976	1.000	0.999	1.000	0.999	0.998	0.979	0.999	0.997
S4	0.998	0.999	0.999	1.000	1.000	0.998	0.997	0.983	0.984	0.998	0.997	1.000	0.996	1.000	0.987	0.996	0.999
S5	0.999	0.999	0.998	1.000	1.000	0.999	0.998	0.987	0.988	0.997	0.995	0.999	0.994	1.000	0.990	0.994	1.000
S6	1.000	1.000	0.995	0.998	0.999	1.000	1.000	0.992	0.992	0.994	0.991	0.997	0.990	0.999	0.994	0.991	1.000
S7	1.000	1.000	0.992	0.997	0.998	1.000	1.000	0.995	0.995	0.991	0.988	0.995	0.987	0.998	0.997	0.987	0.999
S8	0.992	0.991	0.975	0.983	0.987	0.992	0.995	1.000	1.000	0.973	0.967	0.979	0.965	0.987	1.000	0.966	0.989
S9	0.992	0.992	0.976	0.984	0.988	0.992	0.995	1.000	1.000	0.974	0.968	0.980	0.966	0.987	0.999	0.967	0.989
S10	0.994	0.995	1.000	0.998	0.997	0.994	0.991	0.973	0.974	1.000	1.000	0.999	0.999	0.997	0.978	0.999	0.997
S11	0.991	0.992	0.999	0.997	0.995	0.991	0.988	0.967	0.968	1.000	1.000	0.998	1.000	0.995	0.972	1.000	0.994
S12	0.997	0.997	1.000	1.000	0.999	0.997	0.995	0.979	0.980	0.999	0.998	1.000	0.998	0.999	0.984	0.998	0.999
S13	0.990	0.991	0.999	0.996	0.994	0.990	0.987	0.965	0.966	0.999	1.000	0.998	1.000	0.994	0.970	0.999	0.993
S14	0.999	0.999	0.998	1.000	1.000	0.999	0.998	0.987	0.987	0.997	0.995	0.999	0.994	1.000	0.990	0.995	1.000
S15	0.994	0.994	0.979	0.987	0.990	0.994	0.997	1.000	0.999	0.978	0.972	0.984	0.970	0.990	1.000	0.972	0.992
S16	0.991	0.991	0.999	0.996	0.994	0.991	0.987	0.966	0.967	0.999	1.000	0.998	0.999	0.995	0.972	1.000	0.994
Sr.	1.000	1.000	0.997	0.999	1.000	1.000	0.999	0.989	0.989	0.997	0.994	0.999	0.993	1.000	0.992	0.994	1.000

Table S4. Principal components initial eigenvalue and contribution rate.

Principal Components	Eigenvalues	Contribution Rate (%)	Cumulative Contribution Rate (%)
A1	6.843	40.252	40.252
A2	4.447	26.161	66.413
A3	1.902	11.189	77.602
A4	1.452	8.543	86.145

Table S5. Factor loading matrix after rotation.

Peak No.	Principal Components			
	**1**	**2**	**3**	**4**
A1	-0.316	0.612	0.572	-0.012
A2	0.249	0.333	0.061	-0.829
A3	0.365	-0.653	0.525	-0.315
A4	0.725	-0.020	-0.194	0.355
A5	0.241	-0.788	0.073	0.406
A6	-0.502	0.370	0.405	0.243
A7	0.400	-0.875	0.141	-0.108
A8	0.356	-0.120	0.790	-0.041
A9	0.484	-0.824	0.087	0.188
A10	0.693	-0.507	0.171	-0.204
A11	0.825	0.163	-0.258	-0.311
A12	0.842	0.423	0.186	0.097
A13	0.857	0.442	-0.048	0.031
A14	0.905	0.372	0.014	0.143
A15	0.273	0.604	0.514	0.289
A16	0.901	0.312	-0.165	0.025
A17	0.940	0.245	-0.111	0.117

1. Validation of methodology

According to the Pharmacopeia guidelines of the People's Republic of China (2020 Version), the validation of HPLC methodology was performed with linear regression analysis and tests of precision, repeatability, stability, and recovery rate.

1.1. Linear relationships

Each standard curve was obtained by plotting the PA (y) against the mixed standard solution concentrations (x, $\mu g \cdot mL^{-1}$). The results are shown in Table S6. The six ingredients each had an excellent linear relationship within their respective detection ranges and the linear coefficient of determination (r^2) exceeded 0.999.

1.2. Precision, repeatability, stability, and recovery tests

Method precision and repeatability were evaluated via successive analyses of six replicates of the same powder sample. The relative standard deviation (R.S.D.) was 0.03-1.66% for retention time (t_R) and 1.11-2.75% for the PA of six characteristic peaks. These findings showed that the method had good repeatability and precision. The stability of the sample solutions was determined by analysing samples at 0, 2, 4, 8, 16, and 24 h after solution preparation. The R.S.D. was 0.04-1.80% for t_R and 1.61-2.89% for the PA of the characteristic peaks. These results showed that the sample solution had good stability within 24 h. The recovery rate was determined by the standard addition method. The recovery of the six standards ranged from 98.01% to 101.92%. The R.S.D. was 1.48-2.61% for PA. The results of the precision, repeatability, stability, and recovery tests are shown in Table S7 and Table S8.

Table S6. Linear relationships table of six active ingredients.

Components	Regression Equation	R2	Linear Range (ug·mL^{-1})
Rosmarinic acid	y = 15,878,93 4.4174 x + 9,901.6548	0.9998	0.72-715.00
Salvianolic acid B	y = 12,215,141.0899 x - 313,767.3676	0.9996	3.36-3,360.00
Dihydrotanshinone I	y = 38,953,496.8495 x - 53,097.2660	0.9990	0.09-94.50
Cryptotanshinone	y = 57,800,973.6466 x - 90,945.4410	0.9990	0.10-102.50
Tanshinone I	y = 36,295,424.6970 x + 3,077.9435	0.9997	0.14-135.50
Tanshinone IIA	y = 62,899,832.6455 x - 170,731.2788	0.9990	0.22-215.00

Table S7. Analytical results of precision, repeatability of six ingredients.

Components	Precision (n=6)		Repeatability (n = 6)	
	RSD of relative Retention time (%)	RSD of Relative Retention Area (%)	RSD of Relative Retention Time (%)	RSD of Relative Retention Area (%)
Rosmarinic acid	0.47	2.75	1.52	2.89
Salvianolic acid B	1.66	1.18	1.80	2.68
Dihydrotanshinone I	0.28	1.45	0.04	2.61
Cryptotanshinone	0.07	1.44	0.07	1.81
Tanshinone I	0.04	1.20	0.05	1.61
Tanshinone IIA	0.05	1.11	0.04	1.89

Table S8. Analytical results of stability and recovery tests of six ingredients.

Components	Stability (n = 6)		Recovery rate (n = 6)	
	RSD of Relative Retention Time (%)	RSD of Relative Retention Area (%)	Recovery Rate (%)	RSD of Retention Area (%)
Rosmarinic acid	0.10	1.70	98.01	1.48
Salvianolic acid B	1.06	1.73	101.56	1.95
Dihydrotanshinone I	0.03	1.76	101.92	2.25
Cryptotanshinone	0.06	1.35	100.63	2.61
Tanshinone I	0.05	1.64	98.27	2.44
Tanshinone IIA	0.10	1.31	100.75	2.39
